# Dry Gangrene in Children with Typhoid Intestinal Perforation: A Report of Two Cases

**DOI:** 10.1155/2018/7097014

**Published:** 2018-10-08

**Authors:** Lofty-John C. Anyanwu, Aminu M. Mohammad, Lawal B. Abdullahi, Mustapha U. Ibrahim, Aliyu U. Farinyaro, Mohammed S. Aliyu, Stephen K. Obaro

**Affiliations:** ^1^Paediatric Surgery Unit, Department of Surgery, Aminu Kano Teaching Hospital, Bayero University Kano, Nigeria; ^2^Orthopaedic Surgery Unit, Department of Surgery, Aminu Kano Teaching Hospital, Bayero University Kano, Nigeria; ^3^Department of Paediatrics, Aminu Kano Teaching Hospital, Kano., Nigeria; ^4^International Foundation Against Infectious Diseases in Nigeria (IFAIN), Nigeria; ^5^Division of Pediatrics Infectious Disease, University of Nebraska Medical Center, Omaha, USA

## Abstract

Intestinal perforation is a life-threatening complication of typhoid fever commonly seen in developing countries, but extraintestinal complications are infrequently reported. We report herein two cases of gangrene seen in children managed for typhoid intestinal perforation, highlighting the challenges faced in their management.

## 1. Introduction

*Salmonella enterica serovar Typhi*, the causative organism in typhoid fever, is a gram-negative facultative anaerobic bacteria which commonly causes intestinal and systemic pathologies in the host [1]. Injuries caused by this organism on the intestinal mucosa result in the systemic translocation of the organism and other intestinal microbiota and may result in intestinal perforation [2]. Being a systemic pathogen, a number of complications have been described in infected patients, although cases of gangrene are rarely reported [3–9]. We include in this report two cases of gangrene seen in children managed for typhoid intestinal perforation in our unit.

## 2. Case Presentation

### 2.1. Case 1

A 9-year-old girl was presented to our hospital on referral from a peripheral hospital with a one-week history of darkish discoloration of both feet and her left hand and a six-day history of a purulent feculent discharge from an exploratory laparotomy wound. She had presented to the referring hospital eight days earlier, with a two-week history of high-grade fever and a one-week history of a generalized abdominal pain and diarrhea. She had an exploratory laparotomy at the referring hospital. Intraoperative findings include a feculent intraperitoneal collection and a single perforation on the antimesenteric border of the terminal ileum. An intraoperative diagnosis of perforated typhoid enteritis was made, and the perforation was closed in two layers. One unit of blood was transfused postoperatively. On the first day of postsurgery, the patient developed darkish discoloration of the left hand and both feet which were associated with pain. She was not a known sickle cell disease patient and had no past histories suggestive of intermittent claudication. On the second day of postoperation, a purulent discharge from the lower aspect of the laparotomy wound was noted, and there was darkening of the feet and duskiness of the distal one-third of both legs. Due to the progressive worsening of the patient's condition, she was referred to our hospital on the seventh day of postoperation.

**Figure 1:**
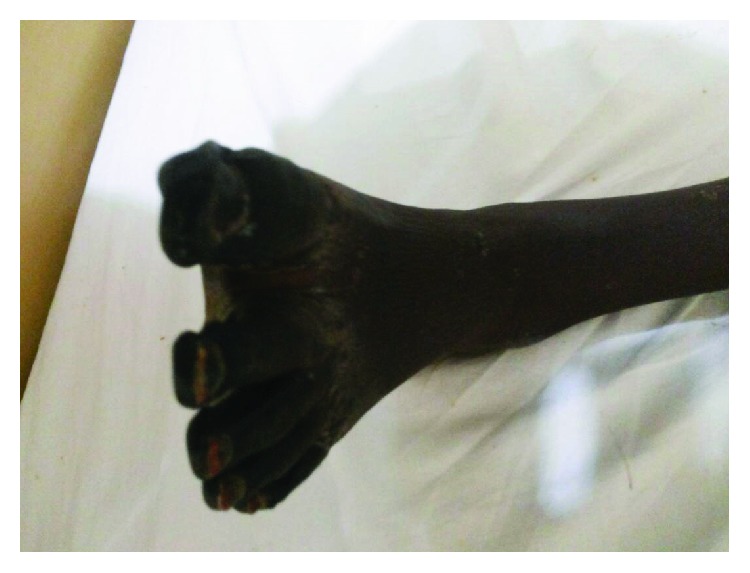
Dry gangrene of the left foot.

At presentation to our unit, she was acutely ill looking, febrile with a temperature of 38.1°C, dehydrated, not pale, and anicteric. Her respiratory rate was 30/minute with reduced air entry and coarse crepitations in both lower lung fields. She had a pulse rate of 130/minute, which was regular and of moderate volume. Only first and second heart sounds were heard. Her abdomen was full, and did not move with respiration, and had a midline dressing soaked with a feculent fluid. There was a complete wound dehiscence of the abdominal wound, with both edges being necrotic. There was no bowel evisceration. Both feet were dark and cold, with duskiness of the skin of the distal one-third of both legs (Figure 1). There was loss of sensation in the feet and absent dorsalis pedis, anterior and posterior tibial pulses bilaterally. Popliteal pulses were palpable bilaterally. The left hand was dark, dry, and shriveled up to the wrist. The radial pulse was however present. The right upper limb was normal. A diagnosis of complete wound dehiscence, fecal fistula with peripheral gangrene of both feet and left hand in a patient who had surgery for typhoid intestinal perforation, was made. The patient was commenced on intravenous (IV) fluid resuscitation and intravenous antibiotics (ceftriaxone and metronidazole). She was placed on nil per os (NPO) and nasogastric decompression of the stomach. A consult was sent to the orthopedic surgery team who advised that the gangrene be allowed to demarcate before any decision could be taken on the limbs.

Hematological investigations after fluid resuscitation showed a hematocrit of 23.9% and a white blood cell count of 18.9 × 10^9^/l and a hemoglobin genotype of AS. She was transfused with 200 ml of packed cells, in preparation for a wound exploration. She was however noted to be making scanty urine, and due to her unstable clinical condition, the surgery was postponed while IV fluid resuscitation and local wound care continued. By the fourth day of admission in our unit, the left upper limb gangrene had extended to the distal forearm, while on the lower limbs, the gangrene had extended to the middle aspect of the legs. The patient's condition continued to deteriorate in spite of efforts at resuscitation. She died on the eighth day of admission in our unit.

### 2.2. Case 2

A 4-year-old boy was presented to our unit on referral with a three-week history of high-grade fever and a five-day history of generalized abdominal pain and abdominal distension. There was an associated history of headaches and body weakness. He had no history of jaundice. He had several episodes of vomiting which was initially none bilious, but later became bilious. There was an associated history of passage of diarrhea stools two days before presentation, although the patient had not passed stools on the day of presentation. He had no history of passage of melena or of hematochezia. His parents complained that he had been passing scanty urine for about five days before presentation. He had been receiving medications from the referring hospital for about two weeks before presentation.

At presentation, he was chronically ill looking with a toxic facie, febrile (temperature 38.5°C), pale, dehydrated, anicteric, and had no pedal edema. His respiratory rate was 28/minute; he had reduced air entry on both lung bases posteriorly. His pulse rate was 128/minute, which was regular but of small volume. Only first and second heart sounds were heard. Abdominal examination showed a distended abdomen which did not move with respiration. He had generalized tenderness with guarding. The bowel sounds were absent. A digital rectal examination showed an empty rectum with a full and tender rectovesical pouch. An initial assessment of a generalized peritonitis was made. The patient was placed on NPO with nasogastric tube for gastric decompression. He was commenced on IV fluid resuscitation and broad spectrum IV antibiotics (ceftriaxone and metronidazole) and was worked up for surgery. Initial laboratory investigations showed a hematocrit of 24% and hypokalemia (2.5 mmol/l) and a serum urea of 10 mmol/l. After an initial fluid resuscitation and correction of serum potassium, he had 300 ml of whole blood transfused. He had an exploratory laparotomy on the second day of admission. At surgery, about 600 ml of a feculent peritoneal fluid was drained, and a single perforation on the antimesenteric border of the terminal ileum was closed in two layers. An intraoperative diagnosis of perforated typhoid enteritis was made. Blood and tissue cultures were not done. He was continued on IV fluids and the same empiric antibiotics after the surgery.

**Figure 2:**
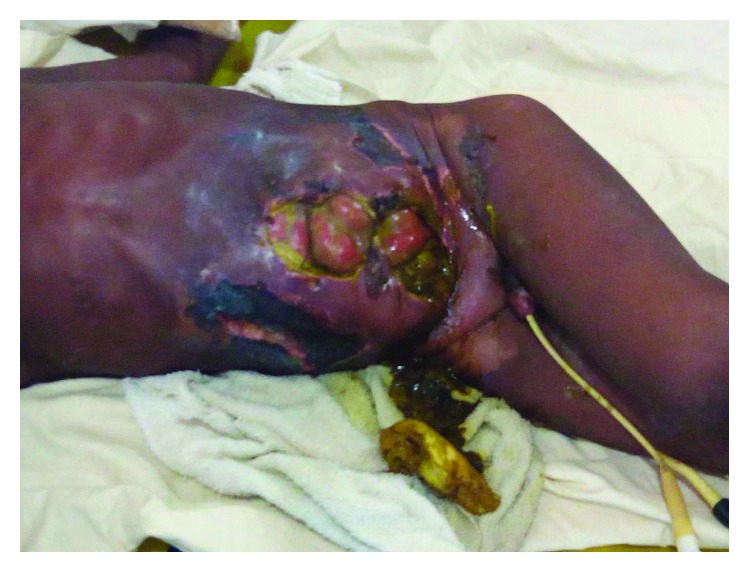
Anterior abdominal wall gangrene with complete wound dehiscence.

On the third day of postoperation, the abdominal wound had a purulent discharge from the distal end. The wound was opened and stitches were removed from the site of drainage, with the institution of daily wound dressing. By the fifth day of postsurgery, a dark patch was noticed to have developed in the skin over the right iliac fossa and measured about 5 cm in its widest diameter. At about the 7^th^ day of postoperation, the dark dry patch had extended to the right hypochondrium, and a similar patch had appeared along the edges of the abdominal incision and the left iliac fossa. A diagnosis of anterior abdominal wall gangrene was made. On the 8^th^ day of postsurgery, the patient was noticed to have developed a complete wound dehiscence (Figure 2) and a fecal fistula. He had fluid resuscitation and was taken back to the theatre on the 10^th^ day after the first surgery, for wound exploration and debridement of the anterior abdominal wall gangrene. A new intestinal perforation was seen at about 2 cm from the initial perforation. The intestinal perforation was exteriorized as an ileostomy, and the abdominal wound closed with tension sutures. Postoperatively, the patient's condition remained unstable, with intractable shock. He died six days after the reexploration of septic shock.

## 3. Discussion

Symmetrical peripheral gangrene (SPG) typically is characterized by ischemic damage and gangrene of two or more distal body parts, without a concomitant occlusion of the principal arterial supply [7, 10, 11]. Although the exact pathogenesis of gangrene in SPG is not known, it may include the release of bacterial endotoxin, the Schwartzman reaction, and sludging of platelets and occlusion of peripheral arterioles in cases due to disseminated intravascular coagulopathy (DIC) and vascular collapse [7, 8, 10, 11]. Only a few cases of gangrene complicating *Salmonella* infections have been reported in literature [7–9]. This report presents two cases of both complications presenting in children with typhoid fever.

In patients with SPG, a low cardiac output state (e.g., shock) together with a hypercoagulable syndrome and a vasospastic condition may coexist, thus leading to occlusion of the microvascular circulation [8, 10, 11]. In SPG, there is a sparing of large vessels with the peripheral pulses usually being palpable [7, 10, 11]. In our first patient, peripheral pulses were already absent at presentation due to rapid progression of the gangrene. Although the etiopathogenesis of occlusion of blood vessels in SPG is not well elucidated, it is not likely to be due to septic emboli, as the gangrene (which is usually dry) starts distally and may progress proximalwards to affect the whole limb or extremity [7, 9, 10]. Also, it has been documented that biopsy specimen taken from gangrenous segments shows microthrombi in both the deep and superficial vascular plexuses, with deposits of fibrin in the lumen and no evidence of inflammatory infiltrates or vasculitis in the wall of the vessels [10].

In *Salmonella* septicemia, the complex lipopolysaccharides in the outer membrane of the bacteria may act as endotoxin which may result in the immune-mediated activation of the fibrinolytic and coagulation pathways and the release of vasoactive factors [5, 8]. Also, DIC has been proposed as the common pathway in SPG pathogenesis [10, 11]. Yoo et al. had earlier reported a case of SPG in a patient with *Klebsiella pneumoniae* sepsis, which was associated with the presence of antiphospholipid antibodies [12]. Both of our patients had septicemia and septic shock, but none of them had any clinical evidence of DIC. None of the patients had a clotting profile or antiphospholipid antibody assay done. Also, the diagnosis of typhoid intestinal perforation in these patients was made on the basis of clinical history of fever followed by an abdominal pain and the intraoperative findings of intraperitoneal soiling and an oval perforation in the long axis of the antimesenteric border of the ileum, which was acutely inflamed and edematous [13]. Similar reports of patients with SPG without DIC have been published in literature [7, 8].

Most reports show that SPG carries a high mortality and morbidity rate, as no particular therapeutic option has been shown to consistently prevent the progression of the gangrene [10, 11]. Early identification of the disease and promptly addressing the known precipitating factors may improve outcome [11]. The presence of anemia, malnutrition, and dehydration has been reported to adversely impact outcome [8]. These features were present in both of our patients in whom the gangrene progressed in spite of institution of intravenous broad spectrum antibiotics, i.e., IV ceftriaxone 50 mg/kg for 12 hours and IV metronidazole 7.5 mg/kg for 8 hours and fluid resuscitation. We believe that endotoxemia and peripheral vascular collapse resulting from shock may have been responsible for the gangrene in our patients. Symmetrical peripheral gangrene usually starts as profound coldness, cyanosis, and pallor of the distal extremity or body part [10]. Some differential diagnoses of this condition include cold agglutinin disease, thromboangiitis obliterans, diabetic gangrene, and thromboembolic gangrene [7, 10, 14]. There were no clinical features of any of these in our patients, although we did not do the Coombs test in any of them.

The treatment of SPG is aimed at addressing the primary pathology and at halting the progression of the gangrene [10, 11]. Given the relative rarity of the condition, most described treatment regimens have not been verified in a randomized controlled trial [11]. Some suggested first-line therapeutic procedures include early institution of IV antibiotics and IV fluids, stoppage of vasopressors, treatment of DIC, thrombolytic therapy, and use of anticoagulant and or vasodilator (e.g., epoprostenol, a prostacyclin) therapy [10–12]. Amputation of the gangrenous segment is usually delayed until the patient is stable, and the gangrene has demarcated [7, 10, 11]. For our patients, we employed broad spectrum IV antibiotics and IV fluid resuscitation. The adequacy of the fluid resuscitation (and peripheral perfusion) was assessed by using an in-dwelling urethral catheter to monitor the hourly urine output in both patients, aiming to achieve a minimum urine volume of 1–2 ml/kg/h.

Some challenges faced in the management of these patients in a resource constrained setting like ours include a lack of laboratory capacity to isolate the causative organism of the sepsis and late presentation of the patients.

Peripheral gangrene complicating typhoid septicemia is a rare but often deadly complication of the disease. A high index of suspicion is recommended in the detection of this complication in children with typhoid fever. Early presentation may improve outcome.
